# Experimental evidence for a cost of resistance to the fungal pathogen, *Batrachochytrium dendrobatidis*, for the palmate newt, *Lissotriton helveticus*

**DOI:** 10.1186/1472-6785-13-27

**Published:** 2013-07-19

**Authors:** Hamed Cheatsazan, Ana P Lugon Gavinho de Almedia, Andrew F Russell, Camille Bonneaud

**Affiliations:** 1 Station d’Ecologie Expérimentale, USR 2936, 2 Route du CNRS, 09200 Moulis, France; 2 Organism and Population Biology, University of Burgundy, 21000 Dijon, France; 3 Centre for Ecology & Conservation, College of Life and Environmental Sciences, University of Exeter Cornwall Campus, Penryn TR10 9EZ, Cornwall, UK

**Keywords:** Body condition, Cost of immunity, Chytridiomycosis, Emerging infectious disease, Resistance, Secondary sexual traits

## Abstract

**Background:**

*Batrachochytrium dendrobatidis* (*Bd*), the causative agent of chytridiomycosis, is decimating amphibians worldwide. Unsurprisingly, the majority of studies have therefore concentrated on documenting morbidity and mortality of susceptible species and projecting population consequences as a consequence of this emerging infectious disease. Currently, there is a paucity of studies investigating the sub-lethal costs of *Bd* in apparently asymptomatic species, particularly in controlled experimental conditions. Here we report the consequences of a single dose of *B. dendrobatidis* zoospores on captive adult palmate newts (*Lissotriton helveticus*) for morphological and behavioural traits that associate with reproductive success.

**Results:**

A single exposure to ~2000 zoospores induced a subclinical *Bd* infection. One week after inoculation 84% of newts tested positive for *Bd*, and of those, 98% had apparently lost the infection by the day 30. However, exposed newts suffered significant mass loss compared with control newts, and those experimental newts removing higher levels of *Bd* lost most mass. We found no evidence to suggest that three secondary sexual characteristics (areas of dorsal crest and rear foot webbing, and length of tail filament) were reduced between experimental versus control newts; in fact, rear foot webbing was 26% more expansive at the end of the experiment in exposed newts. Finally, compared with unexposed controls, exposure to *Bd* was associated with a 50% earlier initiation of the non-reproductive terrestrial phase.

**Conclusions:**

Our results suggest that *Bd* has measureable, but sub-lethal effects, on adult palmate newts, at least under the laboratory conditions presented. We conclude that the effects reported are most likely to be mediated through the initiation of costly immune responses and/or tissue repair mechanisms. Although we found no evidence of hastened secondary sexual trait regression, through reducing individual body condition and potentially, breeding season duration, we predict that *Bd* exposure might have negative impacts on populations of palmate newts through reducing individual reproductive success and adult recruitment.

## Background

*Batrachochytrium dendrobatidis* (*Bd*), the causative agent of chytridiomycosis, has been implicated in worldwide amphibian declines [1]. Although *Bd* is able to infect a wide range of species and hence displays extreme host generality [2-4], amphibian morbidity and mortality in response to infection is highly variable and host-species specific [5,6]. For example, while some studies report devastating consequences of *Bd* infection [7-9], others have shown different levels of resistance to *Bd* infection (capacity to prevent or clear *Bd* infection) and have provided experimental evidence showing some species are able to clear *Bd* infection [7,10-13]. In several species, infiltration of neutrophils, lymphocytes, macrophages and inflammation have been reported on the skin of susceptible (i.e., those which are not able to clear *Bd* infection or to prevent colonization of *Bd*) amphibians [11,14-16]. Histological examinations of resistant hosts have revealed that *Bd* infections can cause skin injuries similar to those observed in susceptible species, albeit distributed more patchily on the skin [10-12]. These results suggest that those amphibians that are apparently resistance to *Bd* infection might still experience sub-lethal costs but whether any such costs are likely to impair traits associated with reproductive success and survival is unclear.

It is well known that pathogens exert significant cost for the host [17,18]. Costs may be manifest through the host’s energetic and cellular response to the infection [19-21], through the pathogens acquisition of host resources, or through inducing greater host susceptibility to other pathogens [22-24]. As such, hosts might show clinical symptoms and visible damage [24], but even where such effects are lacking, significant negative consequences can arise through reductions in investment in traits associated with fitness (e.g., growth, survival and reproduction, [18,19]). As a consequence, in species exhibiting apparent resistance to *Bd*, we might also expect there to be sub-lethal costs of infection and for such costs to influence populations. In support, a number of studies in frogs and toads have reported subclinical effects of *Bd* on tadpole growth [25-28], as well as adult body size [29] and body condition [30]. Nevertheless, in such cases, tests of the consequences of *Bd* infection for traits important in reproduction (morphology, behaviour) of resistant amphibians are generally lacking.

The palmate newt, *Lissotriton helveticus* (Cuadata: Salamandridae; formerly *Triturus helveticus*), is a common, semi-aquatic amphibian of Western Europe. Palmate newts inhabit aquatic habitats with still or very slow-moving water during the breeding season, but otherwise have a terrestrial life style [31], occupying moist habitats and refugia [32]. Males develop large ornaments when they enter water for breeding, manifested as a low, straight edged crest which extends dorsally, a thread-like filament projecting from the end of the tail and conspicuous webbing between the toes of the hind feet [33]. These secondary sexual characteristics have been shown to be associated with male reproductive success [31,34-36]. They then gradually start to regress as the newts leave their aquatic habitat, but may not necessarily have fully disappeared as individuals start their migration to their terrestrial feeding [33].

Interestingly, palmate newts have been reported in several *Bd* infected sites across Europe and have been found to test positively for *Bd* (global Bd maps, see [37] for details). Despite this, no morbidity or mass mortality have been reported [37,38] and under laboratory conditions, excessive weekly exposures to more than 10^6^ *Bd* zoospores per week for more than 4 weeks does not induce symptomatic chytridiomycosis even up to 40 days following a month-long exposure course (Cheatsazan, unpublished results 2011, n = 35 newts). These observations suggest that this species exhibits some level of resistance to *Bd*. However, the consequences of exposure to *Bd*, which will most likely occur during the aquatic phase as a result of increased probability of exposure to waterborne *Bd* zoospores, have not been investigated for this species. Here, we inoculated a group of wild-caught palmate newts experimentally with a single dose of *Bd* (18 controls and 50 exposed) and monitored *Bd* infection, survival and clinical symptoms, as well as changes in morphological and behavioural traits known to be associated with reproductive success in this species. Our specific aim was to use this experiment to test: (1) whether palmate newts become infected by exposure to low doses of *Bd*, whether they show external symptoms and whether they are able to tolerate or clear the infection; (2) the consequences of *Bd* exposure and infection load for secondary sexual characters in males; (3) the consequences of *Bd* exposure and infection load for mass change in both males and females; and (4) whether exposure to *Bd* is associated with changes in the probability of an early entry into the terrestrial phase. Throughout, we consider sex differences, where possible.

## Methods

In mid May 2011, 68 newts (34 males and 34 females) in aquatic phase were captured in narrow streams on the borders of Bouconne forest, Haute Garonne, France (43° 39’ N and 1° 14’ W, 190 m altitude). Our rationale for using wild-caught rather than laboratory-derived newts was that we wished to use individuals as close as possible to their natural state. The sites used are in a forest reserve with natural and man-made streams, and are located about 100 km outside the current extent of the known *Bd* infection zone. In addition, our inspections for *Bd* in this area since 2009 have failed to detect evidence of *Bd* infection of any amphibian species in the area, and all animals brought back to the laboratory tested negative for *Bd*. Male and female newts were randomly paired and transferred into plastic tanks (205× 205 × 140 mm). Our reason for this is that wished to examine the effects of *Bd* exposure on male secondary sexual characteristics, which we considered to be likely influenced by the presence of a female. Each tank contained 1.0 litre of aged tap water and a hollow brick for shelter. We randomly assigned tanks to one of 2 treatment groups: controls (9 males, 9 females) and *Bd*-exposed (25 males and 25 females). All newts were maintained in the laboratory for 7 days prior to experimentation and were provided with live midge larvae (blood worms), daphnia and/or tubifex every two days. Food was provided by ‘sucking’ a known quantity of liquid (tube length x diameter = 55 cmm x 0.5 cm) containing roughly 75 larvae. Although the precise number of larvae was unknown, there is no reason to suppose this varied systematically between experimentally exposed and control tanks. Room temperature was maintained at 18.6 ± 1.9°C throughout the experiment. Light exposure was adjusted weekly to equal the average day length of the first and of the last days of the focal week (approximately 14 h light: 10 h dark). Water was changed 3 days after inoculation and then weekly thereafter to prevent bacterial blooms in the tank. This also means that levels of *Bd* loads detected were unlikely to be explained by zoospore survival in the water [39].

*Bd* cultures were prepared from a *Bd* extract of an infected tadpole from an introduced population of American bullfrog (*Lithobates catesbeianus*) in southern France (for extraction protocol see [40]). This extract was grown in 1.0% Trypton (SCHARLAU, cat. No 07–119) and 0.32 g × l^-1^ glucose (ROTH, cat. No X997) in ultrapure water. Cultures were incubated at 18.5°C to optimize growth of the fungus [5]. Approximately one hour prior to inoculation, we counted approximately 160 active zoospores × ml^-1^ of the 5th passage of our culture using a haemocytometer. We then added 12 ml of this culture to each *Bd* exposed tank (~2000 zoospores × l^-1^ × tank^-1^). The experiment was stopped 30 days post-inoculation, when about approximately half of the newts had entered or were entering the terrestrial phase of the season (t-phase). When a newt consistently remained out of the water for five consecutive days, we considered the day the newt had left the water for the first time as the start of t-phase. Animal capture was with permission of the prefecture of Haute Garonne (Permission No. 2009–12). Animal housing facility and experiments comply with the regulations of the housing organization (CNRS: National centre for scientific research) and the current rules of France.

Infection was detected by quantitative amplification of *Bd*-DNA content of weekly swab samples (from: forelimbs, hind limbs, abdomen and cloaca) using fine tip dry swabs (Tubed sterile dryswabTM tip, Medical wire & Equipment, cat. No MW100). Swab samples were taken at upon arrival, day 7, 14, 21, and 30 after the first inoculation from all individuals. Quantitative PCR’s were conducted as described in [41], and after incorporating the changes suggested by Kriger et al. and Hyatt et al. [42-44] except that the we reduced the final volume of reactions to 10 μl due to higher sensitivity of our q-PCR machine (Mastercycler ep realplex4, Eppendorf). Throughout this paper, results of all quantifications are presented in *Bd* Genome Equivalent (*Bd*GE) per swab bout. In addition to standard duplicates and negative controls, we also included 6–12 samples of control newts in each test plates of exposed samples and utilized internal positive control reagent in all samples (TaqMan® Exogenous Internal Positive Control). To ensure the efficiency of inoculations, we also analysed swab samples of 66 individuals of the same population, after 2 weekly exposures to 10^6^ zoospores of the same *Bd* strain. The average *Bd* load of these newts, one week after the second inoculation, was (mean ± S.E.) 442.2 ± 187.4 *Bd*GE, showing the *Bd* strain and inoculation method was able to generate an infection in this population.

On days 0 and 30 post-inoculation, we measured body mass using a digital scale (±0.01 gram). Snout-to-vent length (SVL) and male secondary sexual traits were measured by ImageJ software on digital photographs of all individuals: area of tail crest, length of tail filament, and average of webbed area of hind feet (left and right feet) one day before inoculation and day 30 after inoculation, according to [36]. Photographs were taken on a single plastic board with a millimetre scaled area of 2 × 10 cm. All morphological measurements were taken twice by the same person (HC) and their average was used in statistical analyses. In all cases a strong correlation (*r* ≥ 0.90, *p* < 0.001) was observed between replicates. Finally, we recorded survival, and the start of t-phase as well as symptoms of clinical chytridiomycosis [5].

### Infection patterns and symptoms

Changes in the probability of being infected during the experiment were analysed by fitting whether (1) or not (0) a newt was infected on a given day as the response term in generalized linear mixed-effects model (GLMM) with a binomial error structure and logit link function. The binomial denominator and dispersion parameters were 1. Changes to infection loads during the course of the experiment, were analysed using a GLMM with Poisson error structure and log-link function in which load size (numbers of *Bd*GE) was fitted as the response term. In both models, individual subjects’ codes (ID’s) nested within box number were fitted as random terms to account for repeated sampling of each. The SVL and body mass of individuals on day zero were fitted as covariates, while sex, days after the first inoculations (7, 14, 21, 30) and their interaction were fitted as fixed effects. Finally, throughout the experiment we examined the animals for signs to *Bd*-mediated symptoms.

### Secondary sexual characteristics in males

Analysis of change in male secondary sexual characters over the course of the experiment was conducting using multivariate analysis of variance, in order to account for multiple correlated terms of interest (crest area, tail filament length and rear foot webbing: Spearman’s rank correlations between the traits, r_s_ = 0.4-0.86, p < 0.005-0.0001). The changes in each trait were fitted as the response terms, while proportional mass change was fitted as a covariate to account for changes in body condition, and treatment was fitted as a the fixed effect of interest. Because in this case we only used a single measure from a single individual from each box (i.e. the male), there was no need to conduct a mixed model in this analysis.

### Mass loss in males and females

The effect of *Bd* on mass was investigated in two ways. First, an analysis of the effect of treatment on mass change was conducted by fitting mass change between day 0 and 30 as the response term in a general linear model with normal errors. Body length and mass at experimental onset were fitted as covariates, while sex and treatment were fitted as the primary fixed effects of interest, along with their interaction. Second, an analysis of the effect of *Bd-*load change during the course of the experiment on mass change was conducted in the same way, but wherein treatment was swapped for load change among exposed individuals. Because both of these analyses included a male and a female from the same box, it could be argued that such pairings are not independent units. However, initial residual maximum likelihood models fitting box number as a random term showed that the box from which individuals were obtained had a negative component of variance, indicating that the variation within boxes was indistinguishable from that between boxes. We therefore elected for the more parsimonious GLM rather than REML approach, since the results were almost identical and the former allows estimation of variance explained.

### Terrestrial phase

The probability that individuals initiated t-phase within the 30-day experimental period was investigated by fitting whether (1) or not (0) individuals entered t-phase as the response term using two GLMs with binomial error structures with logit link functions. In the first case, treatment was fitted as the primary factor of interest, and in the second, treatment was replaced by *Bd*-load. In each case, body length (SVL) and mass at experimental onset were fitted as covariates, and sex was fitted as an additional factor. In both analyses, we investigated the effect of tank identity in GLMMs, but in both cases it represented a negative component of variance.

All statistics were conducted in Genstat Release 14 (Rothamsted Experimental Station, Harpenden, UK). The statistics are provided for all terms included in the models, and effect sizes ± standard errors are provided for terms of interest. Terms were dropped from models when their exclusion failed to generate a significant loss of model variance. All p values provided are two-tailed.

## Results

### Infection patterns and symptoms

**Figure 1:**
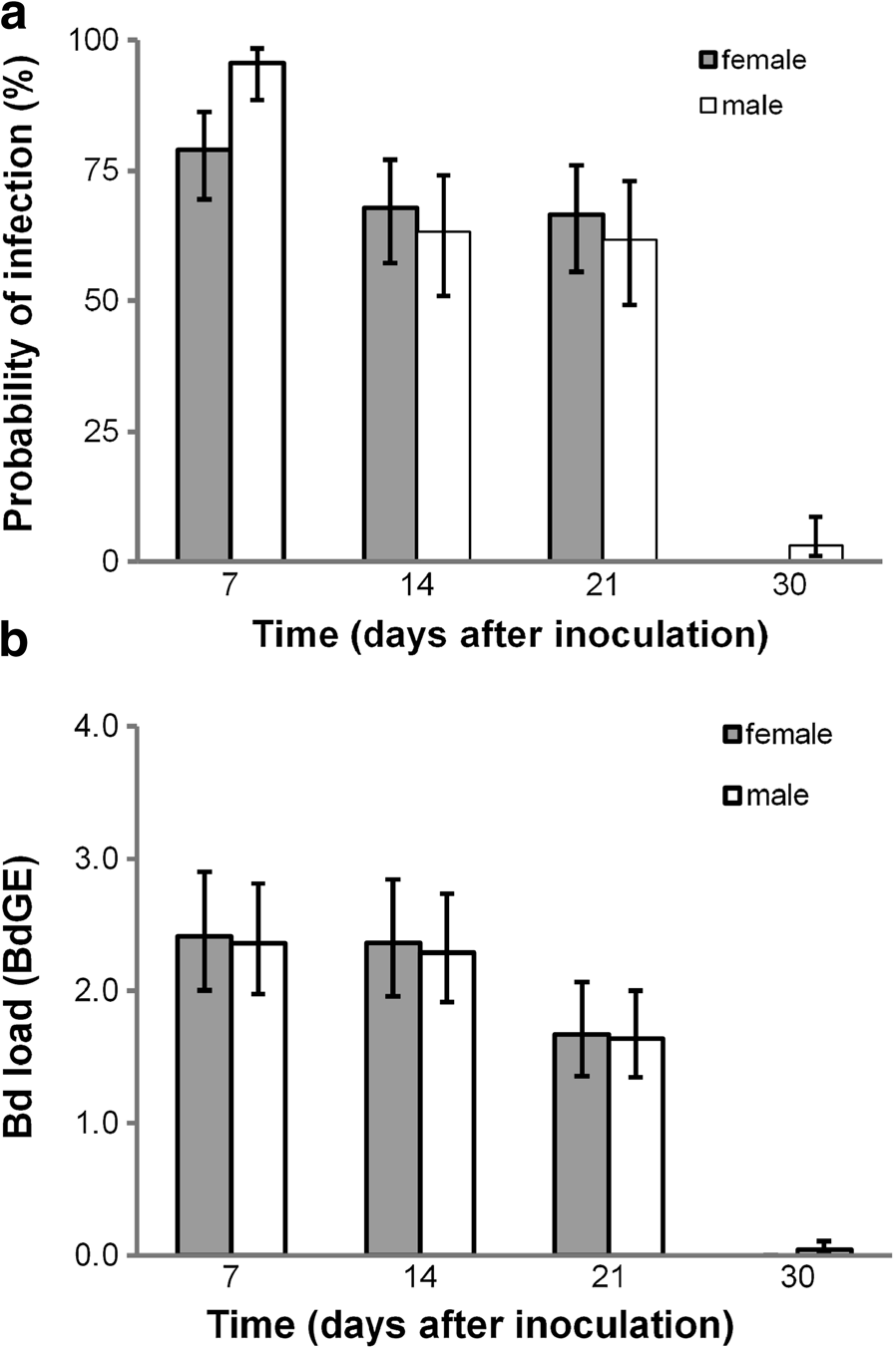
**Changes in probability and intensity of the infection during the course of the experiment. (a)** The probability of infection gradually decreased after the first week and by 30 days post-inoculation, most newts tested negative for *Bd* (GLMM: days post-inoculation; χ^2^ = 30.12, df = 3, p < 0.0001; interaction sex x days post-inoculation χ^2^ = 2.68, df = 3, p = 0.44)*.***(b)** The average infection burden was constant until 2 weeks after inoculation; during the third week after inoculation the infection intensity started to decline and generally reached zero at day 30 post-inoculation (GLMM: days post-inoculation; χ^2^ = 9.20, df = 3, p < 0.001; interaction sex x days post-inoculation χ^2^ = 0.01, df = 3, p = 0.99). Figures show predicted means ± SE generated from the mixed model analyses. In both analyses, tank had no influence on patterns of *Bd* infection (tank component = 0), but there was some consistency in the prevalence of *Bd* infection within versus among individuals (component = 0.01 ±0.009 (S.E.)) and a significant difference between individuals in *Bd* loads (component = 0.12 ±0.004 (S.E.)).

At experimental onset, all newts tested negative for *Bd*, but following exposure to *Bd*, 94% of individuals became infected. Of these, most became infected in the first week, although 4% became infected by the end of week two and 2% were found to be infected at the end of week three. Only 4% of newts remained infected 30 days after the onset of the experiment. As a consequence, the probability that individuals were infected with *Bd* changed substantially during the course of the month-long experiment (Figure 1a). Larger newts were less likely to be infected on a given day (effect ± S.E. = −0.20 ± 0.097; χ^2^ = 4.16, df = 1, p = 0.041), but there was no effect of initial body mass (effect ± S.E. = −0.73 ± 1.46 χ^2^ = 0.49, df = 1, p = 0.49) and no differences between the sexes (χ^2^ = 0.15, df = 1, p = 0.70; Figure 1a) on patterns of infection.

Over the course of the experiment, *Bd* loads ranged from 0–14 *Bd*GE per swab. Newts that were long (SVL) had reduced loads on average (GLMM with Poisson errors, effect ± S.E.; -0.075 ±0.035; χ^2^ = 4.70, df = 1, p = 0.030), but there was no effect of body mass at the onset of the experiment (0.42 ± 0.44; χ^2^ = 0.90, df = 1, p = 0.34) and no difference between the sexes (χ^2^ = 0.01, df = 1, p = 0.93, Figure 1b). As with the incidence data above, *Bd* loads varied dramatically throughout the month-long experiment: in both sexes, *Bd* loads were at their maximum seven days after inoculation and declined to zero after a month (Figure 1b).

The results above suggest that palmate newts are resistant to *Bd*. In support, we found no evidence to suggest that *Bd* induces mortality or visible clinical symptoms in the captive palmate newts. During the course of our 30-day study, only three newts died. Two infected newts died (1 male and 1 female) 15 days post-inoculation, and one control (a female) did so 24 days following the onset of the experiment. Prior to their death, none of the exposed newts showed symptoms of clinical chytridiomycosis, such as skin lesions, haemorrhage, or absence of righting reflex [45,46]. *Bd* contents of swab samples taken the day before of death of the two treated newts were 0 and 14 *Bd*GE in female and male individual, respectively.

### Secondary sexual characteristics in males

**Figure 2:**
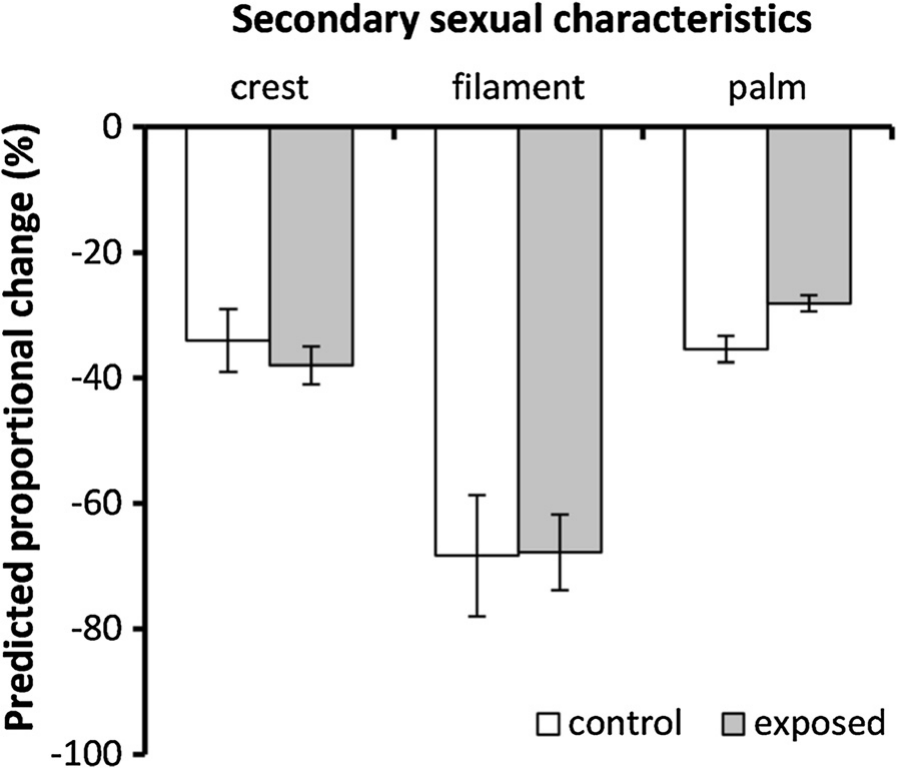
**Effects of *Bd *exposure on male’s secondary sexual traits.** Exposed and non-exposed males showed similar reductions in crest area and tail filament length, but the former showed reduced reabsorption of their rear feet webbing. Figures show predicted means ± S.E. from MANOVA.

Male palmate newts show three obvious morphological characteristics during the aquatic breeding phase. At experimental onset, the mean (±SD) crest area, tail filament length and hind food webbing area were 110.4 (±12.2) mm^2^, 4.6 (±1.2) mm, 18.2 (±1.3) mm^2^, respectively. The onset of the experiment was timed to coincide with the peak breeding season and hence the maximum extent of male secondary sexual characteristic sizes. During the course of the experiment, crest and hind food web area declined by 37% and 31%, respectively, while tail filament was reduced in length by 68% (Figure 2). A multivariate analysis of variance (MANOVA) revealed that after controlling for the proportion of mass lost (F_3,26_ = 3.04, p = 0.047), there was an overall effect of treatment on the reduction of the extent of secondary sexual characteristics (F_3,26_ = 5.43, p = 0.005). However, univariate ANOVAs showed that this overall effect was driven entirely by the effect of Bd treatment on a reduction (not increase) of the loss of hind food web area (crest area, F_1,28_ = 0.45, p = 0.51; filament length, F_1,28_ = 0.01, p = 0.94; hind food web area, F_1,28_ = 12.37, p = 0.002).

**Figure 3:**
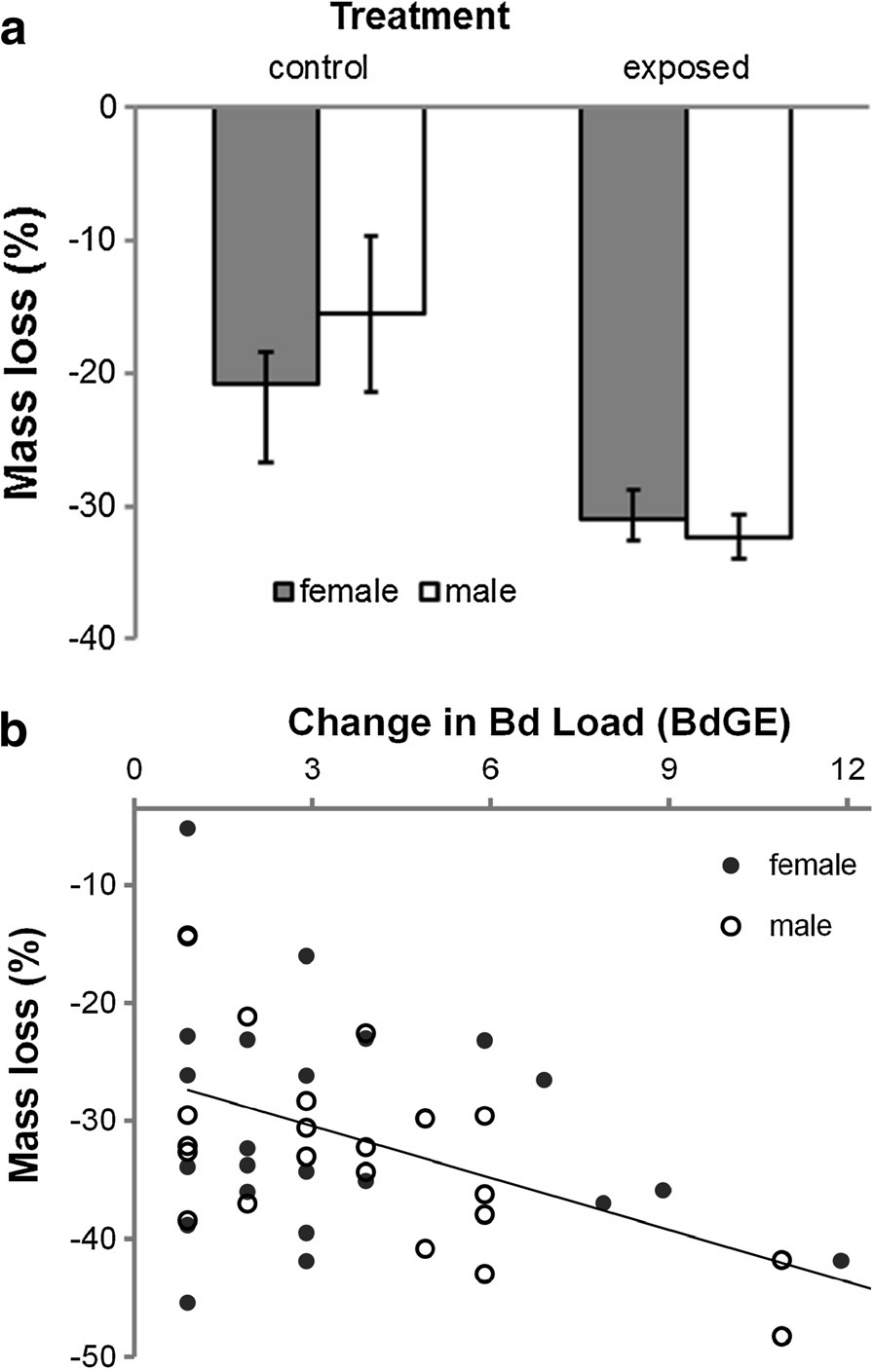
**Effects of *Bd *exposure and load on body mass during the 30-day experiment. (a)** Exposed newts of both sexes lost more mass than unexposed controls; **(b)** among the infected newts, the greater variation in the *Bd* load was correspondent with greater mass loss. Figures show **(a)** predicted means ± S.E. and **(b)** raw values and predicted line.

### Mass loss in males and females

At the time of capture, females were slightly longer than males (mean ± SD snout-vent length (hereafter body length) = 34.9 ± 1.9 *vs*. 32.6 ± 1.8 mm: t-test, t_66_ = 5.10, p < 0.001), but there was no difference in their respective masses (1.19 ±0.23 *vs*. 1.20 ±0.20 g: t_63_ = 0.49, p = 0.63). Individuals that were heavy at experimental onset lost significantly more mass over the 30-day experiment than those that were light (GLM: F_1,63_ = 30.72, p < 0.001), but there was no effect of body length (GLM: effect ± s.e. = −0.0064 ± 0.0058; F_1,62_ = 1.22, p = 0.27). After controlling for significant effects of the former, we found that exposed newts lost 33% more mass than control newts, with treatment explaining 7.5% of the variation in mass change during the experiment (F_1,63_ = 10.55, p = 0.002; Figure 3a). Males and females lost similar amounts of mass (F_1,62_ = 1.23, p = 0.27) and the effect of experimental treatment on mass loss did not differ between the sexes (F_1,61_ = 0.28, p = 0.60; Figure 3a).

**Figure 4:**
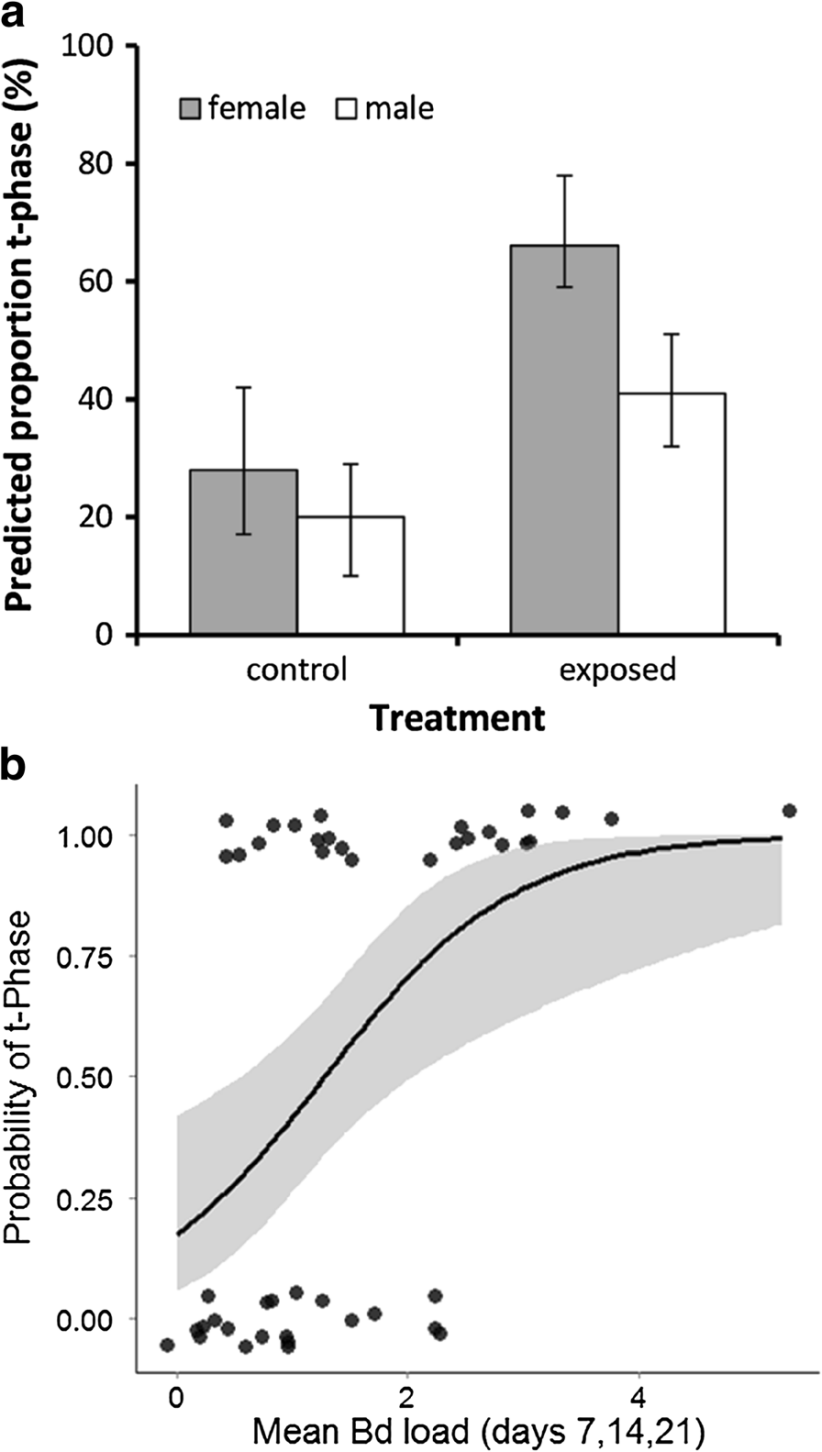
**Consequences of *Bd*-exposure and *Bd*-infection for probability of termination of aquatic breeding season. (a)** Exposed individuals were more likely to enter the terrestrial phase than unexposed controls; and **(b)** higher infection intensities were associated with increased probability of transition to terrestrial phase. Figures show GLM predicted **(a)** predicted percentages from a GLM ± SE and **(b)** shows the relationship between average *Bd* load and occurrence of t-phase among the *Bd* exposed newts. The line represents the linear predictor of the GLM (probability of t-phase) and the shaded area shows ± SE of the predictor.

The results above suggest that palmate newts suffer a cost of clearing *Bd* from their system. However, a better test of this hypothesis is to investigate the relationship between the change in *Bd* load and the change in mass over the course of the experiment. The mass lost by experimental newts increased as a function of increasing body mass at the onset of the experiment (GLM: effect ± S.E. = 0.27 ±0.066; F_1,44_ = 17.61, p < 0.001), but was uninfluenced by SVL (effect ± S.E. = −0.0022 ± 0.0062; F_1,43_ = 0.13, p = 0.73). After controlling for the effects of mass at experimental onset, we found a significant negative relationship with *Bd* load change and mass loss, with newts losing 5% of their body mass for every four *Bd*GE’s that they cleared (effect ± S.E. = 0.051 ± 0.012; F_1,44_ = 18.36, p < 0.001, R^2^ = 20%; Figure 3b). This effect was common to both males and females (GLM: sex*Bd change interaction; F_1,42_ = 0.03, p = 0.86).

### Terrestrial phase

Overall, 46% of newts were judged to have entered t-phase by the end of the 30-day experiment. There were non-significant tendencies for males to enter t-phase after females (GLM, χ^2^ = 1.31, p = 0.25) and those in poor body condition to enter t-phase before those in good condition (body mass correcting for body length, χ^2^ = 2.56, p = 0.11). After controlling for these effects, we found that exposed newts were 50% more likely to enter into t-phase during the 30-day experiment that control newts (χ^2^ = 3.99, p = 0.046; Figure 4a).

Within the exposed individuals, males and females had a similar probability of entering into t-phase (GLM; Sex, χ^2^ = 1.02, p = 0.31). There was no effect of body length on the probability of entering t phase (SVL effect ± S.E. = 0.027 ± 0.15; χ^2^ = 0.03, p = 0.86), but those individuals in poor conditions were more likely to enter the t-phase than those in better condition (initial mass effect ± S.E.-3.46 ± 1.69; χ^2^ = 4.71, p = 0.030). After controlling for these effects, we found that newts were more likely to enter t-phase if they had high average *Bd* loads during the first three weeks of the experiment, independently of sex (*Bd* load effects ± S.E. = 0.73 ± 0.33; χ^2^ = 5.92, p = 0.015; sex x *Bd* load χ^2^ = 0.01, p = 0.99; Figure 4b).

## Discussion

Our results suggest that the *Bd* dose administered is capable of inducing a subclinical infection in the palmate newt within one to two weeks after inoculation. We found no evidence to suggest that *Bd* caused signs of chytridiomycosis [46] or death, and indeed, virtually all exposed newts appeared to have cleared any infection by one month post-inoculation. These results complement circumstantial field evidence documenting that in *Bd* areas, palmate newts appear not to suffer mass mortality (e.g., [37,38]). Nevertheless, our evidence also suggest that such apparent resistance to *Bd* comes at a cost of increased mass loss during the aquatic phase and a more rapid transition to terrestrial t-phase compared to non-exposed controls. Within exposed newts, both the amount of mass lost and probability of entering t-phase increased as a function of increasing pathogen load clearance. By contrast, the rate of loss of secondary sexual characteristics were generally not influenced by *Bd* infection, with the exception of hind foot webbing that remained longer in exposed newts than controls. While the devastating impacts of *Bd* on amphibians are well publicised [7-9,47], much less is known about the extent, form and underlying causes of more subtle symptoms in apparently resistant amphibian species. Our results suggest that caution should be exercised before concluding that *Bd* has negligible consequences for apparently resistant species.

*Bd* is known to invade the host epidermis; feeding on various nutrients (e.g., keratin), causing pathological abnormalities and impairing critical cutaneous functions, such as the maintenance of osmotic balance (reviewed in [5]). Although *Bd* infection can have devastating consequences (see Introduction), accumulating evidence suggests that some amphibians only exhibit subclinical symptoms and might be able to effectively clear the infection through mechanisms such as antimicrobial peptides [13,48], *Bd* killing microbial flora on their skin [49-51], anti-*Bd* immunoglobulin’s [52-54], increasing body temperature during the infection [55] and improvements to dietary condition [56] (reviewed in [57]). In such circumstances, individuals might still suffer costs: (i) because pathogens impair body functioning; (ii) because mounting an immune response or repairing damaged tissues requires energy; (iii) because pathogens actually consume host energy resources; and/or (iv) because immune-associated illness-induced anorexia reduces energy intake [23,58-60]. For instance, in wild frog populations, *Bd* infection has found to be associated with smaller body size [29,61], although the mechanism(s) causing the reduction in body size in these frog studies was unclear. The evidence for our study suggests that increased mass loss might be mediated by a cost of immunity [22], but verification of this as a specific mechanism needs elucidating through more targeted experimentation in our and other studies. For example, we are not able to rule out a role of adaptive anorexia, but we suggest that such an effect is unlikely to explain our results fully, since newts were not fed *adlib* and we noticed no obvious surplus of food in experimental tanks. Indeed, that a recent study has shown experimentally that mounting an innate immune response (skin peptides) against *Bd* comes at cost to host body condition [62], provides some tentative support for our conclusions.

We found little evidence to suggest that the regression of secondary sexual characteristics were hastened by exposure to *Bd*, but we found some support for the possibility that breeding season duration might be curtailed. Both the regression of sexually selected characteristics and transition into t-phase are thought to be largely under hormonal control [63-65]. Although, we were not able to measure neuroendocrine changes of the exposed and infected newts, our results fit well with the current knowledge of amphibians’ stress responses and its impacts on reproduction. In amphibians, exposure to pathogens can cause a rapid release of anti-microbial peptides [66,67] through activation of hypothalamic-pituitary-adrenal (HPA) axis (=stress axis in mammals) [67-69]. Stimulation of this axis can result in inhibition of production and release of stress hormone (i.e. corticosterone) [70,71] which, in urodeles, inhibits the courtship behaviour, development/maintenance of male secondary sexual traits and triggers the migration toward the terrestrial habitat [63,64,72-74]. At the behavioural level, a decrease of prolactin triggers the termination of aquatic phase and migration toward terrestrial habitat while at a morphological level the decrease of prolactin induces the decline of tail crest [63,64,73,74]. However, the decline of hind feet webbing is mediated by a different mechanism which involves a synergetic effect of several hormones [73,75]. Therefore, the slowed reduction of hind feet webbings, in comparison to tail crest and tail filament, might be due to the difference of the hormonal bases which control these traits. In order to elucidate the potential mechanisms of slowed regression of foot webbing (in comparison with other secondary sexual traits, or vice versa) as well as early entry into t-phase, more studies are required. In addition, in order to understand the potential consequences, the exact role and relative importance of each sexually selected trait in female choice is required, as is the consequences of the size of each trait for survival on transition to the t-phase.

Although, for obvious ethical and conservation reasons, we were unable to measure the consequences of experimental infection for individual fitness in the wild, we suggest the consequences of *Bd* that we observed are likely to be significant [26,29,30,76]. In palmate newts, mating success is likely to be influenced by the duration of their aquatic phase, and is known to be condition-dependent: female fecundity and male display rate are both highly demanding energetically [77-79]. It is also highly probable that the success of terrestrial migrations are at least partly associated with having sufficient energy reserves as is the ability to survive winter hibernation, since the annual rate of survival of newts is fairly low (i.e. ≤ 50%, see [80,81]) and newts consume almost all their resources during the winter [78]. Our ability to project the population consequences of sub-lethal infections requires an understanding of whether or not individuals can acquire adaptive immunity to *Bd* or whether individuals with primed immune system remain susceptible to *Bd*. Where the former is the case, we would expect *Bd* to have little impact on palmate newt’s populations once resistance spreads in the populations (e.g. see [62,82]). On the other hand, if the latter is true, the sub-lethal consequences observed in this study are likely to have more significant population consequences, with possible impairment of female fecundity, juvenile recruitment and adult survival. Currently, it is unclear whether amphibian species that suffer subclinical effects of *Bd* are declining, as one might expect from our results. We urge that future studies are careful to monitor population sizes of all amphibian species in a given area, and attempt to determine whether *Bd* can also have population consequences, even for apparently resistant species. Further, in the advent of such declines being apparent, it is important to determine whether such declines are generated through biased effects on each sex or age class.

## Conclusion

The costs of pathogen infection are common and include a range of sub-lethal symptoms including reduced growth, increased mass loss, increased metabolic rate and/or a readjustment of life history strategies [22,83,84]. Our results suggest that subclinical costs of *Bd* infection can exert significant costs on an apparently resistant host amphibian, with potential threatening consequences on long term population viability. Indeed, *Bd* infected palmate newts suffered from a decrease in body condition relative to controls and had delayed absorption of one temporary adaptations for aquatic life; both of which might impede the success of terrestrial migration. In addition, they showed earlier initiation of t-phase, which might reduce the duration of a breeding season, and hence the number of offspring produced by a given population. Furthermore, in addition to the general inhibitory effects of stress on amphibian reproduction, chronic exposure to *Bd* zoospores in the aquatic habitat may trigger a chronic activation of the stress (HPA) axis which might increase an individual’s susceptibility to other pathogens as well as other environmental stressors. Further studies are required in this species and other apparently asymptomatic species, in order to further appreciate the extent, form and mechanism of sub-lethal costs of *Bd* infection in amphibian species.

## Competing interests

The authors confirm that this work is not a subject of financial and non-financial competing interests.

## Authors’ contributions

HC conceived and conducted the experiments with help from AP. The analyses were performed and the paper was written by HC, AFR and CB. All authors read and approved the final manuscript.
